# Mass spectrometry-based metabolomics reveal the effects and potential mechanism of isochlorogenic acid A in MC3T3-E1 cells

**DOI:** 10.3389/fmolb.2025.1518873

**Published:** 2025-03-25

**Authors:** Lian Zhu, Liu Xie, Ziming Wang, Kai-Lin Li, Wei Cai

**Affiliations:** ^1^ School of Pharmaceutical Sciences, Sino-Pakistan Center on Traditional Chinese Medicine, Hunan University of Medicine, Huaihua, China; ^2^ Department of Pathology and Research Office of the School of Basic Medicine, Hunan University of Medicine, Huaihua, China

**Keywords:** osteoporosis, MC3T3-E1 cells, metabolomics, UHPLC-Q-Exactive Orbitrap MS, *Duhaldea nervosa*, mechanism

## Abstract

**Introduction:**

The bioactive compound 3,5-DiCQA, derived from Duhaldea nervosa, has been traditionally utilized in folk remedies for bone fractures and osteoporosis. However, its therapeutic mechanisms remain unclear.

**Methods:**

We employed UHPLC-Q Exactive Orbitrap MS-based cell metabolomics to investigate the molecular mechanisms of 3,5-DiCQA in MC3T3-E1 cells. Cell proliferation was assessed via MTT assay, differentiation by alkaline phosphatase (ALP) activity, and mineralization through alizarin red staining and cetylpyridinium chloride quantification. Metabolomic profiling compared drug-treated and control groups.

**Results:**

Results from MTT assays demonstrated that 3,5-DiCQA significantly promoted cell proliferation at 100 μM. Alkaline phosphatase (ALP) assays and alizarin red staining revealed enhanced osteoblast differentiation and mineralization, respectively. Calcification deposition was significantly increased in the calcified stained cells by cetylpyridinium chloride quantization, indicating that 3,5-DiCQA can promote the mineralization of MC3T3-E1 cells. Metabolomic analysis identified key metabolic changes, including the downregulation of phytosphingosine and upregulation of sphinganine and citric acid.

**Discussion:**

These findings suggest that 3,5-DiCQA promotes osteoblast proliferation, differentiation and mineralization through pathways such as sphingolipid metabolism, arginine and proline metabolism, mucin type O-glycan biosynthesis and the citrate cycle (TCA cycle). This study provides insights into the therapeutic potential of 3,5-DiCQA for osteoporosis and highlights the utility of metabolomics in elucidating traditional Chinese medicine (TCM).

## 1 Introduction

Osteoporosis (OP) is a chronic, systemic endocrine and metabolic disorder. There are two kinds of osteoporosis primary (caused by aging or a lack of sex hormones) and secondary (caused by hyperthyroidism, diabetes, obesity, Cushing’s syndrome, anorexia, rheumatoid arthritis, drug effects, etc.). The root cause of its occurrence is the imbalance of bone remodeling homeostasis including osteoclasts that absorb old bone and osteoblasts that form new bone. This causes the rate of bone loss to be faster than that of bone production (Föger-Samwald, Dovjak, Azizi-Semrad, Kerschan-Schindl and Pietschmann, 2020; Hardy, Zhou, Seibel and Cooper, 2018; Inaba, 2004; Lademann, Tsourdi, Hofbauer and Rauner, 2020; Mo et al., 2021; NIH Consensus Development Panel on Osteoporosis Prevention, Diagnosis, and Therapy, 2001; Workman, Blalock and Mehler, 2020). Therefore, the proliferation, differentiation and mineralization of osteoblasts play a very important role in fracture healing (Dirckx, Van Hul and Maes, 2013). As the population ages, osteoporosis and osteoporoid-related fractures have become a major public health problem for society and significantly increase the consumption of healthcare resources. Therefore, in-depth study of the pathological mechanism of osteoporosis will help reduce the medical costs associated with osteoporosis, and further targeted drug development can improve the quality of life of the elderly.

*Duhaldea nervosa* (Wallich ex Candolle) A. Anderberg, is a member of the Asteraceae family and is commonly known as Maoxiucai or Xiaoheiyao in China (Cai et al., 2020; Liu et al., 2018; Guan et al., 2017). It has been used as a folk medicine for dispelling wind-chill, fighting inflammation and treating a variety of conditions and diseases including fracture and rheumatoid arthritis (RA) (Long, 2004; Xiao, 2009; Xiao et al., 2013). Previous research has shown that *D. nervosa* contains isochlorogenic acid A (3,5-DiCQA), a chemical that has a wide range of physiological activities, such as cardiovascular protection, antioxidant and anti-inflammatory effects, and osteoblast proliferation, which might have a therapeutic effect in the treatment of fractures and RA (Naveed et al., 2018; Wang and Xiao, 2019). However, there are relatively few reports on the efficacy and metabolic pathways of 3,5-DiCQA in treating osteoporosis. Osteoblasts are bone lining cells responsible for the production of bone matrix components and minerals in the process of bone formation (Florencio-Silva, Sasso, Sasso-Cerri, Simões and Cerri, 2015). The regulation of the activity of MC3T3-E1 osteoblasts is of great significance for the prevention and treatment of fractures (Croucher, McDonald and Martin, 2016; Long, 2011). Therefore, it is of great significance to investigate the regulation of 3,5-DiCQA using an *in vitro* MC3T3-E1 cell model.

Metabolomics is a burgeoning field that emerged as an influential analytical approach for identifying potential biomarkers and unraveling the molecular underpinnings of Traditional Chinese Medicine (TCM) in disease treatment (Cheong, Yu, Chen and Zhong, 2022; Wang et al., 2021). The subfield of cellular metabolomics has garnered extensive interest, proving instrumental in scrutinizing the biochemical processes related to disease pathology. It offers insights into how TCM impacts cellular metabolism, thereby contributing to a comprehensive understanding of metabolic processes. For instance, recent studies have demonstrated the utility of cellular metabolomics in elucidating disease mechanisms, such as mitochondrial dysfunction in hypoxia/reoxygenation injury in cardiomyocytes (Lin et al., 2023) and oxidative stress in HepG2 cells (Yu et al., 2024). They have also uncovered the metabolic reprogramming of immune cells in response to inflammation (Wang et al., 2024). These findings highlight the potential of metabolomics to provide comprehensive insights into cellular metabolism and its role in health and disease. Advanced high-resolution mass spectrometry (HRMS) has solidified its role as the principal analytical platform within metabolomics studies. Its prevalence is due to its capacity for sensitive detection, precise resolution of complex mixtures, high precision in mass measurement, and its broad dynamic range, making it an indispensable asset in the quest to decode the metabolomic signatures of various biological systems (Sun et al., 2018; Xie et al., 2023; Yu et al., 2016; Yu et al., 2017). The union of Ultra-High-Performance Liquid Chromatography (UHPLC) with Q-Exactive Orbitrap Mass Spectrometry (MS) stands out as an exceptionally potent analytical methodology for both detecting and characterizing the chemical constituents within botanical extracts and complex biological matrices. The efficacy of this technique is largely due to the swift and decisive separation capabilities of UHPLC, complemented by the Q-Exactive Orbitrap’s prowess in delivering precise mass measurements coupled with a wealth of detailed fragment ion data from MSn experiments, which are crucial for the elucidation of molecular structures (Cai et al., 2017; Clifford, Johnston, Knight and Kuhnert, 2003; Qiao et al., 2016). This approach has been successfully applied in various studies, such as the investigation of Cynara scolymus Bracts’s effects on liver and breast carcinoma cells (El Sohafy et al., 2024) and the metabolic changes in mitochondrial dysfunction in kidney tubular cells (Marchese et al., 2022), demonstrating its versatility and reliability in cellular metabolomics research. Therefore, we used UHPLC-Q-Exactive Orbitrap MS to investigate the molecular mechanisms of 3,5-DiCQA in MC3T3-E1 cells to elucidate its therapeutic mechanism in osteoporosis.

## 2 Materials and methods

### 2.1 Materials and reagents

3,5-DiCQA was purchased from Chengdu Herpurify Co.,Ltd. Liquid chromatography-mass spectrometry (LC-MS/MS)-grade acetonitrile, LC-MS/MS-grade formic acid and the BCA protein concentration assay kit were purchased from Thermo Fisher Scientific Co., Ltd. Ultra-pure water was obtained from Guangzhou Watsons Food & Beverage Co., Ltd. Other solvents were of analytical grade and were supplied by the Aladdin Industrial Corporation.

Fetal bovine serum (FBS) was acquired from Zhejiang Tianhang Biotechnology Co., Ltd. α-MEM medium, tryptic digestion solution and 100 X penicillin streptomycin solution (containing 10 kU/mL penicillin+10 mg/mL streptomycin) were purchased from Hyclone. Dimethyl sulfoxide (DMSO), β-glycerophosphate sodium, vitamin C, estradiol (E2) and 3-(4,5-dimethyl-2-thiazolyl)-2,5-diphenyl-2-H-tetrazolium bromide (MTT) were bought from Sigma Chemical Co., Ltd. The alkaline phosphatase (ALP) kit was acquired from Nanjing Jiancheng Bioengineering Institute. The BCIP/NBT Alkaline Phosphatase Kit was purchased from Beyotime Biotechnology.

### 2.2 Solution preparation

The compound 3,5-DiCQA was prepared as a stock solution at a concentration of 100 mM in DMSO and stored in a dark environment at −20°C for subsequent use. Prior to experimentation, this stock solution was appropriately diluted with α-MEM medium to achieve the desired working concentrations.

### 2.3 Cell culture

The MC3T3-E1 cell line sourced from the National Collection of Authenticated Cell Cultures was maintained in an incubator at 37°C with an atmosphere containing 5% CO_2_. The culture medium was α-MEM supplemented with 10% FBS, 100 units/mL of penicillin, and 10 mg/mL of streptomycin. Upon reaching 80% confluence, the cells were passaged, sub-cultured, and then cryopreserved for future use.

### 2.4 Cell proliferation assay

The MTT assay was utilized to evaluate the viability of MC3T3-E1 cells. The cells were seeded in 96-well plates at a density of 5 × 10^3^ cells per well. To determine the impact of 3,5-DiCQA on osteoblast viability, they were exposed to various concentrations of 3,5-DiCQA (12.5, 25, 50, and 100 μM) for both 24 and 48 h. Following incubation, 10 μL of MTT solution was added to 90 μL of complete medium and the cells were returned to the CO_2_ incubator for an additional 4 h. The absorbance was measured at a wavelength of 490 nm using a microplate reader (Biotek).

### 2.5 ALP activity and staining assay

The influence of 3,5-DiCQA on osteogenic differentiation was investigated by treating experimental groups with different concentrations of 3,5-DiCQA (25, 50, and 100 μM) alongside 10 nM estradiol. After a 6-day incubation period, ALP activity was quantified using a commercial ALP Assay Kit. The microplate reader was set to a wavelength of 562 nm for detection. Additionally, ALP staining was performed using the BCIP/NBT ALP Kit to visualize the activity.

### 2.6 Mineralization assay

The extent of mineralization was assessed using alizarin red staining. MC3T3-E1 cells were cultured in osteogenic induction medium, which contains 50 μg/mL of ascorbic acid and 10 mM β-glycerophosphate, and treated with varying concentrations of 3,5-DiCQA (12.5, 25, 50, and 100 μM) along with 10 nM estradiol for a period of 14 days. The cells were then stained with alizarin red S for 30 min to visualize mineralization nodules. The stained nodules were photographed, and 10% cetylpyridinium chloride (CPC) was utilized to extract the alizarin red for quantification, with the detection wavelength set to 540 nm.

### 2.7 Cell metabolomics

#### 2.7.1 Cell sample collection and preparation

The MC3T3-E1 cells were cultured in 24-well plates and treated with 3,5-DiCQA for a period of 6 days. After incubation, the cells were meticulously rinsed with phosphate-buffered saline (PBS) three times. A volume of 1 mL of chilled methanol was then added to each dish, followed by gently scraping the cells using a cell scraper while on ice. The cells underwent a freeze–thaw cycle three times to facilitate extraction. The mixture was centrifuged at 4°C with a rotation speed of 12,000 rpm for 20 min to collect the supernatant. The supernatant was carefully transferred into LC-MS vials and conserved at −80°C for future analysis. To ensure the reliability of the LC-MS system and to mitigate potential bias, a quality control (QC) sample was crafted. The injection sequence was designed such that a QC sample was interspersed every five samples.

#### 2.7.2 UHPLC-orbitrap-HRMS analysis

For the UHPLC-Orbitrap-HRMS analysis, the cell samples were processed using a Q-Exactive Focus Orbitrap mass spectrometer (Thermo Electron, Bremen, Germany), interfaced with a Thermo Scientific Dionex Ultimate 3000 RS liquid chromatography system (Thermo Fisher Scientific, California, United States) through an electrospray ionization (ESI) source. The chromatographic separation was achieved using a Thermo Scientific Hypersil GOLDTM aQ column (100 mm × 2.1 mm, 1.9 μm), with the column temperature regulated at 40°C. The mobile phase consisted of 0.1% formic acid in water (phase A) and acetonitrile (phase B), with a flow rate of 0.3 mL/min, according to the following gradient elution program: 0–2 min, 5%–40% B; 2–3 min, 40%–55% B; 3–5 min, 55%–69% B; 5–7 min, 69%–70% B; 7–10 min, 70%–73% B; 10–12 min, 73%–95% B; 12–12.1 min, 95%–5% B; and 15 min, 5% B. The injection volume was 2 μL.

High-resolution mass spectrometry (HRMS) operations were conducted using an ESI ion source, capable of both positive and negative ion detection modes. The spray voltage was set to 3.5 kV for the positive mode and 3.2 kV for the negative mode, with sheath gas pressure at 35 arb and auxiliary gas pressure at 10 arb. The capillary and auxiliary gas heater temperatures were maintained at 320°C and 350°C, respectively, and the S-lens RF level was adjusted to 60. Full scan data acquisition was performed over a mass range of *m/z* 100–1,200, utilizing data-dependent MS2 scanning. Nitrogen was utilized as the collision gas, with the energy set to a normalized collision energy of 30%. The entire system was controlled using Xcalibur software, version 4.2.

#### 2.7.3 Data processing

The raw data underwent comprehensive processing utilizing the Compound Discoverer 3.3 software (Thermo, United States). A strict mass tolerance threshold of 5 parts per million (ppm) was applied. The metabolomics workflow was engaged to dissect the mass spectrometry data. Key parameters for analysis were defined, focusing on peaks with signal intensities exceeding a threshold of 10,000 for identification. A retention time window of 0.1 min and a noise elimination threshold of 10 were implemented. Critical data points including peak identification, m/z values, retention times, and signal intensities were extracted and prepared for use in subsequent experimental phases. SIMCA 14.1 software (Umetrics, Sweden) was used for the multivariate statistical treatment of the data, including principal component analysis (PCA), orthogonal partial least squares discriminant analysis (OPLS-DA), and other advanced statistical techniques. The quality of the OPLS-DA model was meticulously assessed through R^2^Y (cumulative) and Q2 (cumulative) metrics, and a stringent 200 permutation test protocol. The variable important in projection (VIP) score and the p-value from the T-test were pivotal in screening potential biomarkers. Metabolite enrichment and pathway analysis were further conducted using the MetaboAnalyst 5.0 online platform, integrating the potential metabolites for a deeper biological interpretation.

### 2.8 Statistical analysis

We used GraphPad Prism (version 9.0) to perform one-way ANOVA. The data are presented as the mean ± standard deviation, derived from a minimum of three replicates per test condition. Statistical significance was determined by a p-value of less than 0.05.

## 3 Results

### 3.1 3,5-DiCQA promoted MC3T3-E1 cells proliferation

**FIGURE 1:**
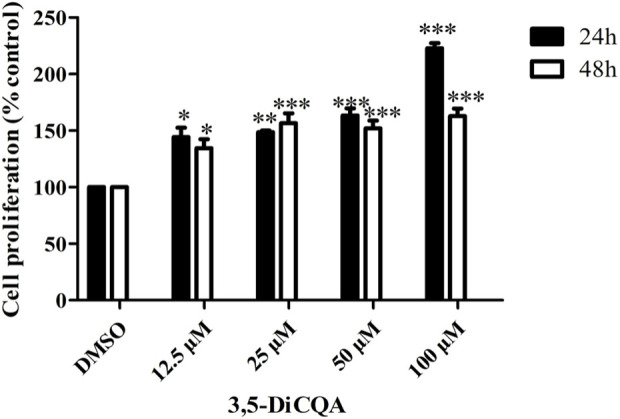
Effects of 3,5-DiCQA at different concentrations on proliferative activity of MC3T3-E1 cells at different time periods. Data were presented as the mean with standard deviation for technical triplicate in an experiment representative of several independent ones (n = 3), *P < 0.05, **P < 0.01, ***P < 0.001, vs. DMSO.

We used an MTT assay to explore the roles of 3,5-DiCQA in the proliferation of MC3T3-E1 cells. The results showed that compared with vehicle treatment, high-dose 3,5-DiCQA (100 μM) significantly promoted cell proliferation in a dose-dependent manner, whereas cell proliferation was significantly reduced 48 h after treatment in MC3T3-E1 cells. As shown in Figure 1, the results indicated that 3,5-DiCQA (12.5–100 μM) significantly promoted cell proliferation (Figure 1).

### 3.2 3,5-DiCQA increased the ALP activity in MC3T3-E1 cells

**FIGURE 2:**
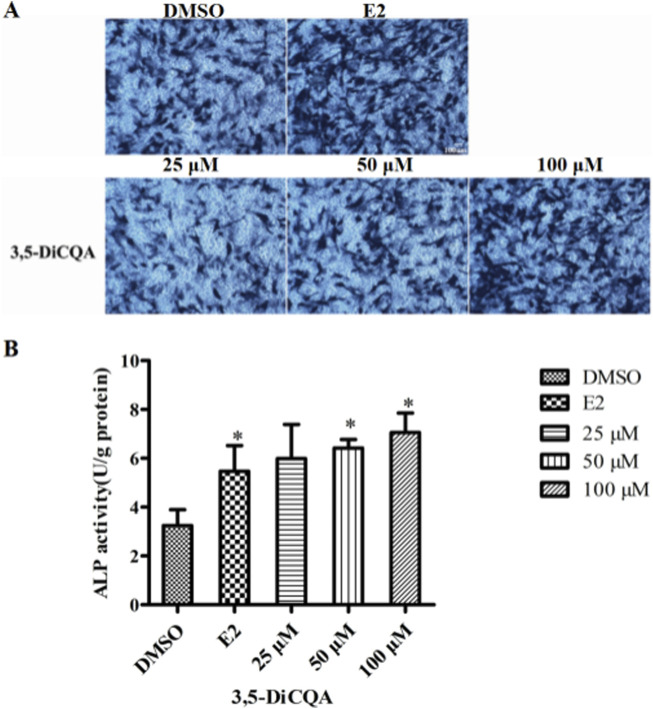
The effect of 3,5-DiCQA on the ALP activity in MC3T3-E1 cells. **(A)** BCIP/NBT staining was conducted. **(B)** The ALP activity was determined after 6-days co-treatment of MC3T3-E1 cells with 3,5-DiCQA (25, 50, and 100 μM) in OIM. Data were presented as the mean with standard deviation for technical triplicate in an experiment representative of several independent ones (n = 5), *p < 0.05 vs. DMSO.

Next, we evaluated whether 3,5-DiCQA would increase the ALP activity in MC3T3-E1 cells. MC3T3-E1 cells were cultured in osteogenic induction medium and incubated with E2 (10 nM) and 3,5-DiCQA (25, 50, and 100 μM) for 6 days. As a result, 3,5-DiCQA significantly increased the ALP activity in MC3T3-E1 cells. Cells treated with high-dose 3,5-DiCQA exhibited stronger ALP staining compared with control cells (Figure 2A). The ALP assay demonstrated that 3,5-DiCQA significantly enhanced cell differentiation (Figure 2B).

### 3.3 3,5-DiCQA increased the mineralization in MC3T3-E1 cells

**FIGURE 3:**
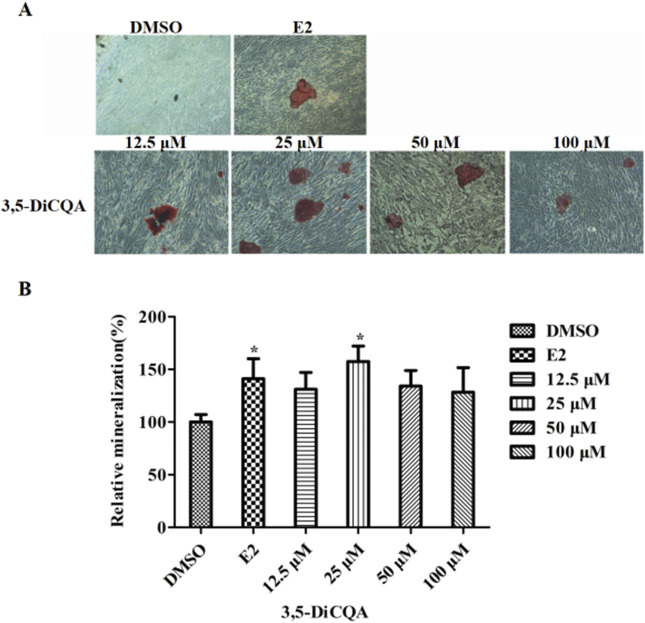
The effect of 3,5-DiCQA on the mineralization of MC3T3-E1 cells. **(A)** Alizarin red S was used for staining on day 14. **(B)** The calcified nodules was quantified by extraction of alizarin red S with 10% cetylpyridinium chloride (CPC) on day 14. Data were presented as the mean with standard deviation for technical triplicate in an experiment representative of several independent ones (n = 6), *p < 0.05 vs. DMSO.

MC3T3-E1 cells were cultured in OIM and incubated with E2 (10 nM) and 3,5-DiCQA (12.5, 25, 50, and 100 μM) for 2 weeks. Alizarin red staining was used to visualize the calcified nodules (Figure 3A). 3,5-DiCQA (12.5, 25, 50, and 100 μM) promoted the formation of calcified nodules in MC3T3-E1 cells. Nodule formation was highest at 3,5-DiCQA treatments of 25 μM (Figure 3B).

### 3.4 Identification of the metabolites of 3,5-DiCQA in MC3T3-E1 cells

From a chemical structure perspective, 3,5-DiCQA is formed by the esterification reaction of two molecules of caffeic acid and one molecule of quinic acid. It may undergo hydrolysis, methylation, sulfation, and other metabolic reactions within cells. By comparing the LC-MS spectra of the control group and the administered group, 11 metabolites (M1∼M11, Table 1) were preliminarily identified from the samples after administration of 3,5-DiCQA. After metabolism, the metabolites retain some basic structural features of the parent drug. Therefore, we can infer the structure of the metabolites by analyzing the mass spectrometry fragmentation patterns of the parent drug 3,5-DiCQA. On comparing the retention time and mass spectrometry data of the standards, the quasi-molecular ion peak of 3,5-DiCQA is *m/z* 515.1195 [M-H]^-^ (with the molecular formula C_25_H_24_O_12_, an error of 0.72), and the retention time is 9.71 min. The MS^2^ spectrum shows the characteristic fragmentation ions resulting from the neutral loss of one molecule of caffeoyl group at *m/z* 353.08 [M-H-caffeoyl]^−^, the neutral loss of two molecules of caffeoyl group at *m/z* 191.05 [M-H-2×caffeoyl]^−^, the neutral loss of one molecule of caffeoyl group and one molecule of quinic acid residue at *m/z* 179.03 [M-H-caffeoyl-quinic acid]^−^, and further neutral loss of one molecule of CO_2_ producing the fragment ion at *m/z* 135.04 [M-H-caffeoyl-quinic acid-CO_2_]^−^. These characteristic fragmentation pathways provide a basis for the identification of metabolites. The retention time of M1 is 3.42 min. Its [M-H]^-^ peak is at m/z 353.08818, which is 162 Da less than m/z 515.1195. It is speculated to be the product formed when 3,5-DiCQA loses one molecule of caffeoyl group during hydrolysis. The double bond on the caffeoyl group of 3,5-DiCQA undergoes a nucleophilic addition reaction with the thiol group of cysteine. After the conjugate metabolic reaction of cysteine, the molecular weight of the product increases by the molecular weight of one cysteine. Therefore, it is speculated that M2, M3 and M5 undergoes the cysteine conjugation metabolic reaction. In the molecule of 3,5-DiCQA, there are carbon-carbon double bonds in the caffeoyl part. These double bonds are the sites where hydration reactions can occur. When 3,5-DiCQA undergoes a hydration reaction once, it is equivalent to adding a water molecule to the molecular structure. Then, the molecular weight of the product M4 after the reaction is the molecular weight of 3,5-DiCQA plus that of a water molecule. By comparing the retention time and MS^2^ spectrum with those of the reference compounds, M8 and M9 were identified as 1,5-DiCQA and 4,5-DiCQA, respectively, both of which are products of the intramolecular acyl migration reaction of 3,5-DiCQA.The retention time of M10 is 11.13 min. The [M-H]^-^ peak is at m/z 529.13515, which is 14 Da higher than m/z 515.1195. This is speculated to be the methylated product of 3,5-DiCQA. The retention time of M12 is 12.56 min. The [M-H]- peak is at m/z 543.15080, which is 28 Da higher than m/z 515.1195. We speculated that this is the dimethyl product of 3,5-DiCQA.

**TABLE 1:** The retention time and mass spectrometric data of isochlorogenic acid A metabolites.

Peak	t_R_	Theoretical Mass *m/z*	Experimental Mass *m/z*	Error (ppm)	Formula	MS/MS fragment (−)	Identification	LYA + Cell	DMSO + Cell	LYA-cell
M1	3.42	353.08781	353.08829	1.37	C_16_H_18_O_9_	MS^2^[353]:191.0555(100), 179.0343(75), 135.0441(23)	Hydrolyzation	+	−	+
M2	5.39	634.12359	634.12476	1.83	C_28_H_29_NO_14_S	MS^2^[634]:191.0556(100), 179.0347(71), 192.9961(41), 353.0901(37), 206.4759(21)	Cysteine Conjugation 1	+	−	−
M3	5.97	636.13924	636.14093	2.64	C_28_H_31_NO_14_S	MS^2^[636]:191.0556(100), 353.0878(72), 179.0344(64), 173.0449(12)	Cysteine Conjugation 2	+	−	−
M4	7.54	533.13006	533.13086	1.49	C_25_H_26_O_13_	MS^2^[533]: 135.0439(100), 173.0447(79), 179.0344(52), 191.0554(31), 335.0781(29)	Hydration	+	−	−
M5	7.91	634.12359	634.12457	1.53	C_28_H_29_NO_14_S	MS^2^[634]:173.0447(100), 192.9958(74), 179.0345(64), 191.0554(45)	Cysteine Conjugation 1	+	−	+
M6	9.63	586.10246	586.10278	0.53	C_27_H_25_NO_12_S	MS^2^[586]:173.0446(100), 250.0177(51), 179.0339(31), 161.0232(23), 335.0789(17)	Dehydration + Dehydration+ Taurine conjugation	+	−	+
M7	9.71^a^	515.11950	515.11987	0.72	C_25_H_24_O_12_	MS2[515]:173.0454(100); 179.0350(87); 191.0561(44); 135.0441(16); 353.0878(15)	3,5-DiCQA	+	−	+
M8	9.88^a^	515.11950	515.11981	0.60	C_25_H_24_O_12_	MS^2^[515]:191.0555(100), 179.0343(80), 353.0878(15), 173.0448(14), 135.0441(14)	1,5-DiCQA	+	−	+
M9	10.70^a^	515.11950	515.11993	0.84	C_25_H_24_O_12_	MS^2^[515]:173.0448(100), 179.0343(72), 191.0555(22), 353.0882(20)	4,5-DiCQA	+	−	+
M10	11.13	529.13515	529.13574	1.12	C_26_H_26_O_12_	MS^2^[529]:193.0497(100), 173.0445(65), 179.0341(58), 161.0234(22), 155.0341(21), 135.0444(17)	methylate	+	−	−
M11	12.56	543.15080	543.15131	0.94	C_27_H_28_O_12_	MS^2^[543]:173.0447(100), 193.0499(85), 175.0392(38), 349.0932(34), 155.0341(29)	Dimethylate	+	−	−

^a^ Confirmed with standard compounds.

### 3.5 Multivariate statistical analysis

**FIGURE 4:**
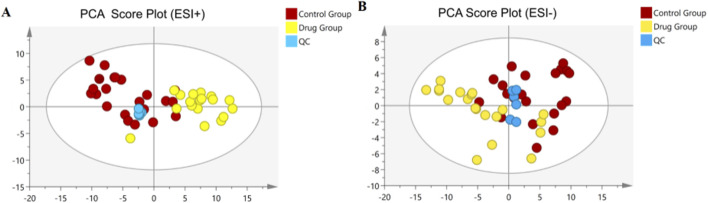
Multivariate statistical analyses of metabolites in MC3T3-E1 cells. **(A)** The PCA score plots in positive modes. **(B)** The PCA score plots in negative modes.

**FIGURE 5:**
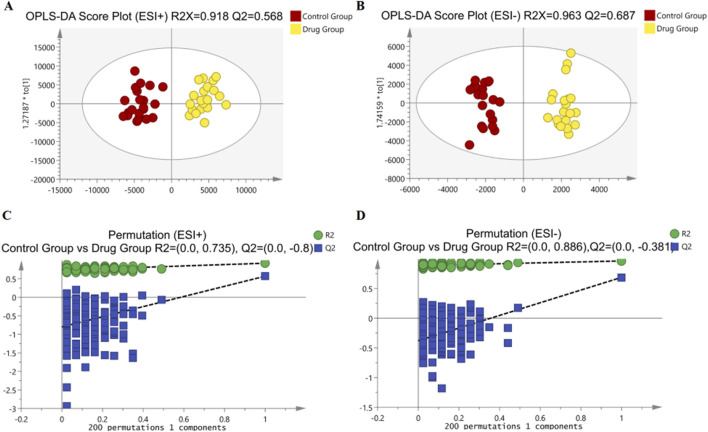
Multivariate statistical analyses of metabolites in MC3T3-E1 cells. **(A, B)** The OPLS-DA score plots comparing Control groups and Drug groups in positive and negative modes, respectively. **(C)** Permutation plot for Control groups and Drug groups by the 200-response reciprocity test in the positive ion mode. **(D)** Permutation plot for Control groups and Drug groups by the 200-response reciprocity test in the negative ion mode.

To delve deeper into the mechanisms by which 3,5-DiCQA influences the differentiation of MC3T3-E1 cells, a comparative metabolite analysis was conducted. We compared the differentiation induced by 3,5-DiCQA with that of cells treated with DMSO using UHPLC-HRMS. An unsupervised PCA approach was employed to assess the general sample distribution and the clustering of quality control (QC) samples as depicted in Figures 4A, B. The PCA plots demonstrated a coherent grouping of the QC samples, with some overlap between the control (DMSO) and the experimental (3,5-DiCQA) groups, which reflects the high stability and methodological soundness of the analytical instrument. In an effort to enhance the differentiation between the control and experimental groups and to boost the model’s analytical resolution, a supervised OPLS-DA analysis was conducted, as presented in Figures 5A, B. The OPLS-DA score plots revealed a significant divergence between the control and experimental groups in both positive and negative modes. This separation confirms the presence of metabolic differences, suggesting that 3,5-DiCQA induces changes in cellular metabolism. To ensure the robustness of the OPLS-DA model against overfitting, a 200 permutation test was applied. The R2Y (cumulative) metric indicates the model’s explanatory power along the y-axis, while the Q2 (cumulative) signifies its predictive accuracy. A Q2 value exceeding 0.5 is generally considered a threshold for model stability and reliability. In this study, the positive ion mode exhibited R2Y and Q2 values of 0.918 and 0.568, respectively, and the negative ion mode showed R2Y and Q2 values of 0.963 and 0.687, respectively. The permutation test results, as illustrated in Figures 5C, D, affirmed the model’s reliability and precision in both ionization modes.

### 3.6 Identification of potential biomarkers

**FIGURE 6:**
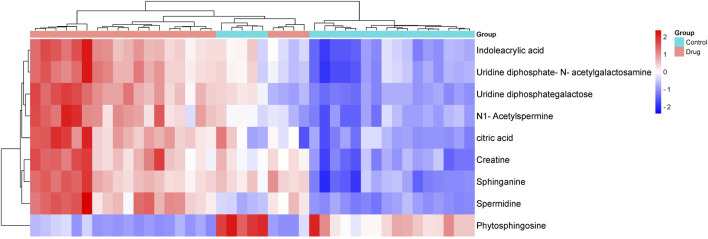
The hierarchically clustered heatmap of metabolite levels in control and drug group. The columns represent samples in different experimental conditions, and the rows represent different biomarkers. Different colors represent the concentration differences of different samples.

Differential metabolite analysis among the groups was performed using multivariate and univariate statistical analysis. Specifically, the OPLS-DA model and t-test were employed to identify variations in metabolite levels. A VIP score exceeding 1, coupled with a t-test p-value below 0.05, were established as thresholds for the significance of differential metabolites. As a result, nine potential biomarkers (Supplementary Figures S1, S2) as detailed in Table 2. A heatmap with hierarchical clustering was used to make data visualization more intuitive. The changed patterns in metabolite concentrations across samples can clearly be seen in Figure 6. A similar color distribution was observed within each group, along with a large difference between the groups. In comparison to the control group, treatment with 3,5-DiCQA led to noticeable decreases in the levels of phytosphingosine while it induced substantial increases in sphinganine and citric acid, as outlined in Table 2. These findings underscore the substantial metabolic alterations induced by 3,5-DiCQA.

**TABLE 2:** The identification of potential biomarkers in MC3T3-E1 cells in the negative and positive ion mode.

t_R_	*m/z*	Formula	Metabolite names	VIP	Average peak area of drug group	SEM of drug group	Average peak area of control group	SEM of control group	p-values	Ion forms	Control vs. drug
0.75	565.04706	C_36_H_10_O_6_N_2_	Uridine diphosphategalactose	3.01	2810689.1	21577.498	1107863.165	154628.5691	1.31912E-07	[M-H] -	↑
0.75	146.16490	C_7_H_19_N_3_	Spermidine	1.85	24053.25838	1091.032746	10364.58899	439.0869989	2.46336E-14	[M + H] +	↑
0.77	245.23306	C_12_H_28_ON_4_	N1-Acetylspermine	2.45	35665.66051	1217.196513	16099.51865	800.6653578	8.37824E-08	[M + H] +	↑
0.84	132.07657	C_4_H_9_O_2_N_3_	Creatine	3.26	61262.51753	1879.515717	20585.75808	1870.734819	1.28547E-09	[M + H] +	↑
0.93	606.07379	C_38_H_13_O_6_N_3_	Uridine diphosphate-N-acetylgalactosamine	4.80	30105.19026	920.4109192	11329.62448	885.2335823	2.58392E-08	[M-H] -	↑
1.16	191.01859	C_6_H_8_O_7_	citric acid	4.70	408605.7443	7815.019972	147799.5476	6114.505469	3.22437E-07	[M-H] -	↑
2.72	188.07025	C_11_H_9_O_2_N	Indoleacrylic acid	2.04	62483.37221	2886.459908	35612.17412	2614.062037	2.4323E-08	[M + H] +	↑
5.68	318.29916	C_18_H_39_O_3_N	Phytosphingosine	2.38	101084.8017	5858.04475	276446.8166	1787.99431	6.41044E-12	[M + H] +	↓
6.90	302.30466	C_18_H_39_O_2_N	Sphinganine	6.41	223552.1397	2809.639479	83680.75176	4208.499282	7.82594E-10	[M + H] +	↑

### 3.7 Metabolic pathway analysis

**FIGURE 7:**
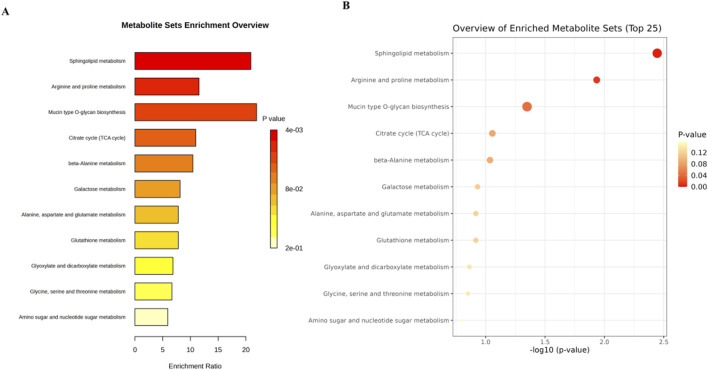
Analysis of metabolic pathway associated with the 3,5-DiCQA promotes MC3T3-E1 cells differentiation using an enrichment analysis with an online MetaboAnalyst 5.0. **(A)** Metabolic pathway enrichment analysis. **(B)** Metabolic pathway topology analysis.

We conducted an in-depth analysis to uncover the metabolic pathways that may be influenced by 3,5-DiCQA in enhancing the differentiation of MC3T3-E1 cells. Utilizing the MetaboAnalyst 5.0 platform, we enriched and examined the topological aspects of 29 metabolic pathways represented by biomarkers. In our graphical representation, the vertical axis denotes the name of the metabolic pathway, while the horizontal axis reflects the enrichment ratio, which is the proportion of altered metabolites relative to the entire pool within a given pathway. Our findings indicated a total of 11 pathways that are potentially modulated by 3,5-DiCQA to facilitate cell differentiation, with notable pathways including sphingolipid metabolism, arginine and proline metabolism, mucin type O-glycan biosynthesis, and the citrate cycle (TCA cycle), as depicted in Figure 7A. In the network topology analysis diagram, each circle symbolizes a distinct metabolic pathway. The variations in the size and color of these circles correspond to the extent of their influence within the system. As illustrated in Figure 7B, the differentiation of MC3T3-E1 cells induced by 3,5-DiCQA appears to be particularly linked to sphingolipid metabolism and several other pathways, the details of which are compiled in Table 3.

**TABLE 3:** Pathway analysis of biomarkers using MetaboAnalyst 5.0 online.

Pathway name	Match status	Expect	p	Holm p	FDR
Sphingolipid metabolism	2/21	0.0957	0.00359	0.301	0.301
Arginine and proline metabolism	2/38	0.173	0.0116	0.9961	0.486
Mucin type O-glycan biosynthesis	1/10	0.0456	0.0448	1.0	1.0
Citrate cycle (TCA cycle)	1/20	0.0911	0.0878	1.0	1.0
beta-Alanine metabolism	1/21	0.0957	0.092	1.0	1.0
Galactose metabolism	1/27	0.123	0.117	1.0	1.0
Alanine, aspartate and glutamate metabolism	1/28	0.128	0.121	1.0	1.0
Glutathione metabolism	1/28	0.128	0.121	1.0	1.0
Glyoxylate and dicarboxylate metabolism	1/32	0.146	0.137	1.0	1.0
Glycine, serine and threonine metabolism	1/33	0.15	0.141	1.0	1.0
Amino sugar and nucleotide sugar metabolism	1/37	0.169	0.157	1.0	1.0

## 4 Discussion

Osteoporosis (OP), characterized by low bone mass, degeneration of bone tissue and destruction of bone microstructure, can lead to decreased bone strength and increased risk of fracture. The number of OP hip fractures worldwide is estimated to exceed 200 million, and 40% of postmenopausal women and 30% of men will experience OP fractures during their lives (Garvey et al., 2016; Wright et al., 2014). In China, the incidence of OP is as high as 23.9% among people between 50 and 59 years old, and the incidence increases significantly with an increase in age (Liao et al., 2002). However, the current treatment of OP includes drug therapy, physical therapy and exercise therapy, but the therapeutic effect is relatively low, patients' compliance is poor, and there are many adverse reactions (Aaseth, Boivin and Andersen, 2012; Metcalf, Aspray and McCloskey, 2017; Piemonte et al., 2012). At present, the clinical treatment of osteoporosis is still dominated by chemical drugs. According to their different mechanisms of action in the treatment of osteoporosis, chemical drugs can be divided into bone absorption inhibitors (such as bisphosphonates, estrogen and calcitonin), bone formation promoters (fluoride and strontium preparations) and bone mineralization promotion drugs (vitamin D and calcium preparations) (An et al., 2016; Zeng et al., 2014; Du et al., 2013; Xu et al., 2018). However, taking these drugs is often accompanied by side effects such as inflammation of the esophagus, nausea, abdominal pain and even cancer of the reproductive system. Their potential toxicity and side effects limit their wide application to some extent (Black et al., 2013; Ma and Ge, 2017). Therefore, the search for safer natural substitutes of traditional Chinese medicine (TCM) that can promote bone formation and reverse bone structural damage is receiving increasing attention.

*Duhaldea nervosa* is traditionally used for activating meridians, promoting blood circulation and removing blood stasis, reducing swelling and dispersing blood. It has a good therapeutic effect on rheumatic pain, fall injury, fracture and other diseases, and can significantly shorten the course of fracture healing. Since ancient times, *Duhaldea nervosa* has been widely used as a medicine for treating fall injury by the Dong people (Long, 2004; Long et al., 2013; Wang et al., 2008; Wang et al., 2009; Zhu and He, 2011). It is common in Dong medicine to mix the stem powder of *Duhaldea nervosa* with glutinous rice sweet distiller’s grains and apply it to the injury or fracture, which can relieve pain, reduce swelling, disperse silting and promote fracture healing. According to our previous experimental studies, chlorogenic acids, especially 3,5-DiCQA are the main component of *Duhaldea nervosa*.

3,5-DiCQA is a dicaffeinoquinic acid found among coffee picolinic acids. The quinic acid component of coffee is a class of natural compounds formed by acidification of quinic acid and varying amounts of coffee. Modern pharmacological studies have shown that dicaffeoylquinic acid has antioxidant, anti-inflammatory, anti-microbial and other pharmacological effects (Fiamegos et al., 2011; Könczöl et al., 2012; Park et al., 2009). Therefore, in this study, MC3T3-E1 cells were used as the cell model *in vitro* to study its effects on the proliferation, differentiation and mineralization of osteoblasts, clarify the specific mechanism of its promotion of osteoblast differentiation and provide an experimental basis for the basic research of its pharmacodynamic substances in treating fall injury and promoting fracture healing.

To further investigate the mechanism by which 3,5-DiCQA promotes MC3T3-E1 cell differentiation, UHPLC-HRMS was used to compare differential metabolites between the control groups and drug groups, for 100 μM 3,5-DiCQA MC3T3-E1 cells. We concluded that 3,5-DiCQA increased the levels of sphinganine and citric acid and decreased the levels of phytosphingosine, which promotes differentiation in MC3T3-E1 cells. Bone remodeling balance is dynamic and easily stimulated by the external environment including energy metabolism substrates, hormones and growth factors (Shaw and Gravallese, 2016). Osteoporosis is also a systemic disorder of energy metabolism, of glucose and lipid metabolism, of abnormal distribution of fatty acids, and disorder of amino acid content, which are closely related to the occurrence and development of osteoporosis (Chin, Wong, Ekeuku and Pang, 2020; During, Penel and Hardouin, 2015; Martyniak et al., 2021; Su et al., 2019). Focusing on the bone microenvironment, the energy metabolism disorder of osteoblasts and osteoclasts is a key factor in pathogenesis. Cell energy production is mainly dependent on glucose Glycolysis (in the cytoplasm), the tricarboxylic acid (TCA) cycle, and oxidative phosphorylation (OXPHOS) (in mitochondria) are the main pathways by which adenine riboside triphosphate (ATP, the most important high energy phosphate bond compound in the body)is produced (Lee, Guntur, Long and Rosen, 2017). The C-H bonds in the molecular structure of energy substances such as glucose, amino acids and fatty acids contain chemical energy. In the process of oxidation, the C-H bonds are broken to generate CO_2_ and H_2_O, and energy is released at the same time. In the cell, the balance of chemical energy regulates the cascade amplification mechanism of many upstream and downstream molecules, thus controlling the transcription, translation and other processes of genes, and finally realizing the control of various cell phenotypes (Miyazaki et al., 2012; Sabbatinelli et al., 2019).

## 5 Conclusion

This study provides new insights into the mechanism of action of traditional Chinese medicines (TCMs) through a holistic cellular metabolomics approach, and has revealed the potential mechanisms by which 3,5-DiCQA promotes the proliferation, differentiation and mineralization of MC3T3-E1 cells. These findings not only provide a scientific basis for 3,5-DiCQA as a candidate for promoting bone formation, but also offer important references for further research into the application of TCM components in bone tissue engineering. However, this study has some limitations, and the results need to be further validated in animal models to explore the mechanism of 3,5-DiCQA.

## Data Availability

The original contributions presented in the study are publicly available. This data can be found here: https://doi.org/10.5061/dryad.c2fqz61n1.

## Abbreviations

3,5-DiCQA, isochlorogenic acid A; TCM, traditional Chinese medicine; ALP, alkaline phosphatase; CPC, cetylpyridinium chloride; FBS, Fetal bovine serum; MTT, 3-(4,5-dimethyl-2-thiazolyl)-2,5-diphenyl-2-H-tetrazolium bromide; DMSO, Dimethyl Sulfoxide; E2, estradiol; LC-MS/MS, Liquid chromatography-mass spectrometry; CO_2_, carbon dioxide; OIM, osteogenic induction medium; PBS, phosphate-buffered saline; QC, quality control; HRMS, High-resolution mass spectrometry; ANOVA, one-way analysis of variance; PCA, principal component analysis; OPLS-DA, orthogonal partial least squares discriminant analysis; VIP, variable important in projection; TCA cycle: Citrate cycle; OP, Osteoporosis; ES, electrospray ionization.

## References

[B1] Aaseth J. Boivin G. Andersen O. (2012). Osteoporosis and trace elements--an overview. J. trace Elem. Med. Biol. organ Soc. Minerals Trace Elem. (GMS) 26, 149–152. 10.1016/j.jtemb.2012.03.017 22575536

[B2] An J. Yang H. Zhang Q. Liu C. Zhao J. Zhang L. (2016). Natural products for treatment of osteoporosis: the effects and mechanisms on promoting osteoblast-mediated bone formation. Life Sci. 147, 46–58. 10.1016/j.lfs.2016.01.024 26796578

[B3] Black D. M. Bilezikian J. P. Greenspan S. L. Wüster C. Muñoz-Torres M. Bone H. G. (2013). Improved adherence with PTH(1-84) in an extension trial for 24 months results in enhanced BMD gains in the treatment of postmenopausal women with osteoporosis. Osteoporos. Int. 24, 1503–1511. 10.1007/s00198-012-2098-3 22930240 PMC4890154

[B4] Cai W. Guan Y. Zhou Y. Wang Y. Ji H. Liu Z. (2017). Detection and characterization of the metabolites of rutaecarpine in rats based on ultra-high-performance liquid chromatography with linear ion trap-Orbitrap mass spectrometer. Pharm. Biol. 55, 294–298. 10.1080/13880209.2016.1236392 27927077 PMC6130507

[B5] Cai W. Li K. L. Xiong P. Gong K.-Y. Zhu L. Yang J.-B. (2020). A systematic strategy for rapid identification of chlorogenic acids derivatives in Duhaldea nervosa using UHPLC-Q-Exactive Orbitrap mass spectrometry. Arabian J. Chem. 13, 3751–3761. 10.1016/j.arabjc.2020.01.007

[B6] Cheong K. L. Yu B. Chen J. Zhong S. (2022). A comprehensive review of the cardioprotective effect of Marine Algae polysaccharide on the gut microbiota. Foods (Basel, Switz.) 11, 3550. 10.3390/foods11223550 PMC9689188 36429141

[B7] Chin K. Y. Wong S. K. Ekeuku S. O. Pang K. L. (2020). Relationship between metabolic syndrome and bone health - an evaluation of Epidemiological studies and mechanisms Involved. Diabetes, metabolic syndrome Obes. targets Ther. 13, 3667–3690. 10.2147/dmso.s275560 PMC7569044 33116718

[B8] Clifford M. N. Johnston K. L. Knight S. Kuhnert N. (2003). Hierarchical scheme for LC-MSn identification of chlorogenic acids. J. Agric. food Chem. 51, 2900–2911. 10.1021/jf026187q 12720369

[B9] Croucher P. I. McDonald M. M. Martin T. J. (2016). Bone metastasis: the importance of the neighbourhood. Nat. Rev. Cancer 16, 373–386. 10.1038/nrc.2016.44 27220481

[B10] Dirckx N. Van Hul M. Maes C. (2013). Osteoblast recruitment to sites of bone formation in skeletal development, homeostasis, and regeneration. Birth defects Res. Part C, Embryo today Rev. 99, 170–191. 10.1002/bdrc.21047 24078495

[B11] Du C. Y. Hu Z. H. Chen L. DuaN J. H. (2013). Effect of alendronate on bone turnover biomarkers in postmenopausal osteoporosis. Chin. J. Osteoporos. 20, 22–25. 10.3969/j.issn.1006-7108.2014.01.005

[B12] During A. Penel G. Hardouin P. (2015). Understanding the local actions of lipids in bone physiology. Prog. lipid Res. 59, 126–146. 10.1016/j.plipres.2015.06.002 26118851

[B13] El Sohafy S. M. Shams Eldin S. M. Sallam S. M. Bakry R. Nassra R. A. Dawood H. M. (2024). Exploring the ethnopharmacological significance of Cynara scolymus bracts: integrating metabolomics, *in-Vitro* cytotoxic studies and network pharmacology for liver and breast anticancer activity assessment. J. Ethnopharmacol. 334, 118583. 10.1016/j.jep.2024.118583 39013541

[B14] Fiamegos Y. C. Kastritis P. L. Exarchou V. Han H. Bonvin A. M. Vervoort J. (2011). Antimicrobial and efflux pump inhibitory activity of caffeoylquinic acids from Artemisia absinthium against gram-positive pathogenic bacteria. PloS one 6, e18127. 10.1371/journal.pone.0018127 21483731 PMC3070693

[B15] Florencio-Silva R. Sasso G. R. Sasso-Cerri E. Simões M. J. Cerri P. S. (2015). Biology of bone tissue: structure, Function, and factors that influence bone cells. Biomed. Res. Int. 2015, 421746. 10.1155/2015/421746 26247020 PMC4515490

[B16] Föger-Samwald U. Dovjak P. Azizi-Semrad U. Kerschan-Schindl K. Pietschmann P. (2020). Osteoporosis: Pathophysiology and therapeutic options. EXCLI J. 19, 1017–1037. 10.17179/excli2020-2591 32788914 PMC7415937

[B17] Garvey W. T. Mechanick J. I. Brett E. M. Garber A. J. Hurley D. L. Jastreboff A. M. (2016). American association of clinical endocrinologists and American college of endocrinology comprehensive clinical practice guidelines for medical care of patients with obesity. Endocr. Pract. official J. Am. Coll. Endocrinol. Am. Assoc. Clin. Endocrinol. 22 (Suppl. 3), 1–203. 10.4158/ep161365.gl 27219496

[B18] Guan Y. Wang Y. Zhou Y. Wang Y. W. Zheng B. J. Wang L. T. (2017). Determination of lsochlorogenic acid A and lsochlorogenic acid C in Duhaldea nervosa by HPLC. Lishizhen Med. Materia Medica Res. 28, 1032–1034. 10.3969/j.issn.1008-0805.2017.05.004

[B19] Hardy R. S. Zhou H. Seibel M. J. Cooper M. S. (2018). Glucocorticoids and bone: Consequences of Endogenous and Exogenous excess and Replacement therapy. Endocr. Rev. 39, 519–548. 10.1210/er.2018-00097 29905835

[B20] Inaba M. (2004). Secondary osteoporosis: thyrotoxicosis, rheumatoid arthritis, and diabetes mellitus. J. bone mineral metabolism 22, 287–292. 10.1007/s00774-004-0501-7 15221485

[B21] Könczöl A. Béni Z. Sipos M. M. Rill A. Háda V. Hohmann J. (2012). Antioxidant activity-guided phytochemical investigation of Artemisia gmelinii Webb. ex Stechm.: isolation and spectroscopic challenges of 3,5-O-dicaffeoyl (epi?) quinic acid and its ethyl ester. J. Pharm. Biomed. Anal. 59, 83–89. 10.1016/j.jpba.2011.10.012 22079045

[B22] Lademann F. Tsourdi E. Hofbauer L. C. Rauner M. (2020). Thyroid hormone actions and bone remodeling - the role of the Wnt signaling pathway. Exp. Clin. Endocrinol. and diabetes official J. Ger. Soc. Endocrinol. Ger. Diabetes Assoc. 128, 450–454. 10.1055/a-1088-1215 31958849

[B23] Lee W. C. Guntur A. R. Long F. Rosen C. J. (2017). Energy metabolism of the osteoblast: Implications for osteoporosis. Endocr. Rev. 38, 255–266. 10.1210/er.2017-00064 28472361 PMC5460680

[B24] Liao E. Y. Wu X. P. Deng X. G. Huang G. Zhu X. P. Long Z. F. (2002). Age-related bone mineral density, accumulated bone loss rate and prevalence of osteoporosis at multiple skeletal sites in Chinese women. Osteoporos. Int. 13, 669–676. 10.1007/s001980200091 12181627

[B25] Lin C. Sang Q. Fu Z. Yang S. Zhang M. Zhang H. (2023). Deciphering mechanism of Zhishi-Xiebai-Guizhi Decoction against hypoxia/reoxygenation injury in cardiomyocytes by cell metabolomics: regulation of oxidative stress and energy acquisition. J. Chromatogr. B Anal. Technol. Biomed. Life Sci. 1216, 123603. 10.1016/j.jchromb.2023.123603 36652817

[B26] Liu L. Zhang J. Zheng B. Guan Y. Wang L. Chen L. (2018). Rapid characterization of chlorogenic acids in Duhaldea nervosa based on ultra-high-performance liquid chromatography-linear trap quadropole-Orbitrap-mass spectrometry and mass spectral trees similarity filter technique. J. Sep. Sci. 41, 1764–1774. 10.1002/jssc.201701047 29327507

[B27] Long F. (2011). Building strong bones: molecular regulation of the osteoblast lineage. Nat. Rev. Mol. cell Biol. 13, 27–38. 10.1038/nrm3254 22189423

[B28] Long K. E. (2004). On the diagnosis and treatment of bone traumatology in Dong medicine of Long family. J. Med. and Pharm. Chin. Minorities S1, 80–81. 10.16041/j.cnki.cn15-1175.2004.s1.161

[B29] Long K. E. Xiao C. W. Long S. Liu B. Zhang D. D. Zeng S. D. (2013). Study on the treatment technology of bone injury and fracture in Dong Medicine (VI) -- Observation on the clinical effect of 4118 cases of bone injury and fracture treated by Dong Medicine. J. Med. and Pharm. Chin. Minorities 19, 22–24. 10.16041/j.cnki.cn15-1175.2013.05.019

[B30] Long S. (2004). Clinical experience of single Dong medicine “Maoshouchai” in traumatology. J. Med. and Pharm. Chin. Minorities, 231–232. 10.16041/j.cnki.cn15-1175.2004.s1.064

[B31] Ma H. Z. Ge J. R. (2017). Preliminary study on the adverse reactions of traditional Chinese medicine in the treatment of osteoporosis. Chin. J. Osteoporos. 23, 548–554. 10.3969/j.issn.1006-7108.2017.04.027

[B32] Marchese E. Caterino M. Viggiano D. Cevenini A. Tolone S. Docimo L. (2022). Metabolomic fingerprinting of renal disease progression in Bardet-Biedl syndrome reveals mitochondrial dysfunction in kidney tubular cells. iScience 25 (11), 105230. 10.1016/j.isci.2022.105230 36281451 PMC9587000

[B33] Martyniak K. Wei F. Ballesteros A. Meckmongkol T. Calder A. Gilbertson T. (2021). Do polyunsaturated fatty acids protect against bone loss in our aging and osteoporotic population? Bone 143, 115736. 10.1016/j.bone.2020.115736 33171312

[B34] Metcalf L. M. Aspray T. J. McCloskey E. V. (2017). The effects of parathyroid hormone peptides on the peripheral skeleton of postmenopausal women. A systematic review. Bone 99, 39–46. 10.1016/j.bone.2017.03.007 28286298

[B35] Miyazaki T. Iwasawa M. Nakashima T. Mori S. Shigemoto K. Nakamura H. (2012). Intracellular and extracellular ATP coordinately regulate the inverse correlation between osteoclast survival and bone resorption. J. Biol. Chem. 287, 37808–37823. 10.1074/jbc.M112.385369 22988253 PMC3488055

[B36] Mo Y. Lai W. Zhong Y. Hu Z. You M. Du M. (2021). TXNIP contributes to bone loss via promoting the mitochondrial oxidative phosphorylation during glucocorticoid-induced osteoporosis. Life Sci. 266, 118938. 10.1016/j.lfs.2020.118938 33347878

[B37] Naveed M. Hejazi V. Abbas M. Kamboh A. A. Khan G. J. Shumzaid M. (2018). Chlorogenic acid (CGA): a pharmacological review and call for further research. Biomed. Pharmacother. 97, 67–74. 10.1016/j.biopha.2017.10.064 29080460

[B38] NIH Consensus Development Panel on Osteoporosis Prevention, Diagnosis, and Therapy (2001). Osteoporosis prevention, diagnosis, and therapy. JAMA 285 (6), 785–795. 10.1001/jama.285.6.785 11176917

[B39] Park K. H. Park M. Choi S. E. Jeong M. S. Kwon J. H. Oh M. H. (2009). The anti-oxidative and anti-inflammatory effects of caffeoyl derivatives from the roots of Aconitum koreanum R. RAYMOND. Biol. and Pharm. Bull. 32, 2029–2033. 10.1248/bpb.32.2029 19952423

[B40] Piemonte S. Romagnoli E. Bratengeier C. Woloszczuk W. Tancredi A. Pepe J. (2012). Serum sclerostin levels decline in post-menopausal women with osteoporosis following treatment with intermittent parathyroid hormone. J. Endocrinol. investigation 35, 866–868. 10.3275/8522 22842667

[B41] Qiao X. Li R. Song W. Miao W. J. Liu J. Chen H. B. (2016). A targeted strategy to analyze untargeted mass spectral data: rapid chemical profiling of Scutellaria baicalensis using ultra-high performance liquid chromatography coupled with hybrid quadrupole orbitrap mass spectrometry and key ion filtering. J. Chromatogr. A 1441, 83–95. 10.1016/j.chroma.2016.02.079 26952367

[B42] Sabbatinelli J. Prattichizzo F. Olivieri F. Procopio A. D. Rippo M. R. Giuliani A. (2019). Where metabolism Meets Senescence: Focus on Endothelial cells. Front. physiology 10, 1523. 10.3389/fphys.2019.01523 PMC6930181 31920721

[B43] Shaw A. T. Gravallese E. M. (2016). Mediators of inflammation and bone remodeling in rheumatic disease. Seminars cell and Dev. Biol. 49, 2–10. 10.1016/j.semcdb.2015.10.013 PMC4761455 26481971

[B44] Su Y. Elshorbagy A. Turner C. Refsum H. Chan R. Kwok T. (2019). Circulating amino acids are associated with bone mineral density decline and ten-year major osteoporotic fracture risk in older community-dwelling adults. Bone 129, 115082. 10.1016/j.bone.2019.115082 31622772 PMC6925590

[B45] Sun L. Jia H. Ma L. Yu M. Yang Y. Liu Y. (2018). Metabolic profiling of hypoxia/reoxygenation injury in H9c2 cells reveals the accumulation of phytosphingosine and the vital role of Dan-Shen in Xin-Ke-Shu. Phytomedicine 49, 83–94. 10.1016/j.phymed.2018.06.026 30217265

[B46] Wang J. Wu S. Gao H. Yu C. Chen X. Yuan Z. (2024). Integrated metabolomics and network pharmacology analysis to explore pig bile-processed Rhizoma Coptidis and Fructus Evodiae sauce-processed Rhizoma Coptidis in lipopolysaccharide-induced inflammatory response. J. Chromatogr. B Anal. Technol. Biomed. Life Sci. 1243, 124192. 10.1016/j.jchromb.2024.124192 38941716

[B47] Wang Q. Xiao L. (2019). Isochlorogenic acid A attenuates acute lung injury induced by LPS via Nf-κB/NLRP3 signaling pathway. Am. J. Transl. Res. 11, 7018–7026. 31814905 PMC6895519

[B48] Wang Y. Chu F. Lin J. Li Y. Johnson N. Zhang J. (2021). Erianin, the main active ingredient of Dendrobium chrysotoxum Lindl, inhibits precancerous lesions of gastric cancer (PLGC) through suppression of the HRAS-PI3K-AKT signaling pathway as revealed by network pharmacology and *in vitro* experimental verification. J. Ethnopharmacol. 279, 114399. 10.1016/j.jep.2021.114399 34246740

[B49] Wang Y. Qiu S. P. Mei S. M. Zheng L. Yang Y. Y. Liu C. Z. (2008). Research on Pharmacognosy of Mao Xiucai. Huangshi, Hubei Province, China: Shizhen Journal of Traditional Chinese Medicine and Materia Medica Press, 1212–1213. 10.3969/j.issn.1008-0805.2008.05.102

[B50] Wang Y. Xiao C. Y. Tian L. Ouyang C. F. (2009). Research on the quality standards of the medicinal materials of the Dong ethnic medicine Mao Xiucai. J Med and Pharm. Chin Minorities 15 (08), 41–43. 10.16041/j.cnki.cn15-1175.2009.08.041

[B51] Workman C. Blalock D. V. Mehler P. S. (2020). Bone density status in a large population of patients with anorexia nervosa. Bone 131, 115161. 10.1016/j.bone.2019.115161 31765843

[B52] Wright N. C. Looker A. C. Saag K. G. Curtis J. R. Delzell E. S. Randall S. (2014). The recent prevalence of osteoporosis and low bone mass in the United States based on bone mineral density at the femoral neck or lumbar spine. J. bone mineral Res. official J. Am. Soc. Bone Mineral Res. 29, 2520–2526. 10.1002/jbmr.2269 PMC4757905 24771492

[B53] Xiao C. W. (2009). On the distinctive diagnosis and treatment of Dong ethnic medicine. J. Med. and Pharm. Chin. Minorities 15, 2–5. 10.16041/j.cnki.cn15-1175.2009.07.007

[B54] Xiao C. W. Shi G. H. Yang X. Q. (2013). Study on Dong Medicine Treatment of bone injury and fracture Technology (V) -- Dong medicine treatment of bone injury and fracture internal and external application of the list, test, secret recipe. J. Med. and Pharm. Chin. Minorities 19, 28–32. 10.16041/j.cnki.cn15-1175.2013.02.018

[B55] Xie H. Hu M. Yu J. Yang X. Li J. Yu N. (2023). Mass spectrometry-based metabolomics reveal Dendrobium huoshanense polysaccharide effects and potential mechanism of N-methyl-N'-nitro-N-nitrosoguanidine -induced damage in GES-1 cells. J. Ethnopharmacol. 310, 116342. 10.1016/j.jep.2023.116342 36889419

[B56] Xu Y. Y. Zhang K. L. Wei Z. M. Shen B. (2018). Experimental study of the effect of estrogen on bone mineral density and bone metabolism in osteoporotic rats. Chin. J. Osteoporos. 24, 776–780. 10.3969/j.issn.1006-7108.2018.06.014

[B57] Yu C. Xu Y. Zhao M. Song P. Yu J. (2024). New insights into mechanism of ellagic acid alleviating arsenic-induced oxidative stress through MAPK/keap1-Nrf2 signaling pathway response, molecular docking and metabolomics analysis in HepG2 cells. Ecotoxicol. Environ. Saf. 285, 117029. 10.1016/j.ecoenv.2024.117029 39277998

[B58] Yu M. Cui F. X. Jia H. M. Zhou C. Yang Y. Zhang H. W. (2016). Aberrant purine metabolism in allergic asthma revealed by plasma metabolomics. J. Pharm. Biomed. Anal. 120, 181–189. 10.1016/j.jpba.2015.12.018 26744988

[B59] Yu M. Jia H. Zhou C. Yang Y. Zhao Y. Yang M. (2017). Variations in gut microbiota and fecal metabolic phenotype associated with depression by 16S rRNA gene sequencing and LC/MS-based metabolomics. J. Pharm. Biomed. Anal. 138, 231–239. 10.1016/j.jpba.2017.02.008 28219800

[B60] Zeng Y. Li Q. He R. (2014). The comparison research of Calcium and calcium joint vitamin D intervention in the treatment of osteoporosis in older men. J. Clin. Exp. Med. 13, 625–629. 10.3969/j.issn.1671-4695.2014.08.007

[B61] Zhu Y. He A. N. (2011). Research on Mao Xiucai's Materia Medica and modern research progress. J. Med. and Pharm. Chin. Minorities 17, 36–38. 10.16041/j.cnki.cn15-1175.2011.10.032

