# Supplementary material for: Mass spectrometry-based metabolomics reveal the effects and potential mechanism of isochlorogenic acid A in MC3T3-E1 cells
Source: Front Mol Biosci. 2025 Mar 25;12:1518873. doi: 10.3389/fmolb.2025.1518873 (PMC11975594; doi:10.3389/fmolb.2025.1518873)
Supplement: Supplementary file 1 [file DataSheet1.pdf]

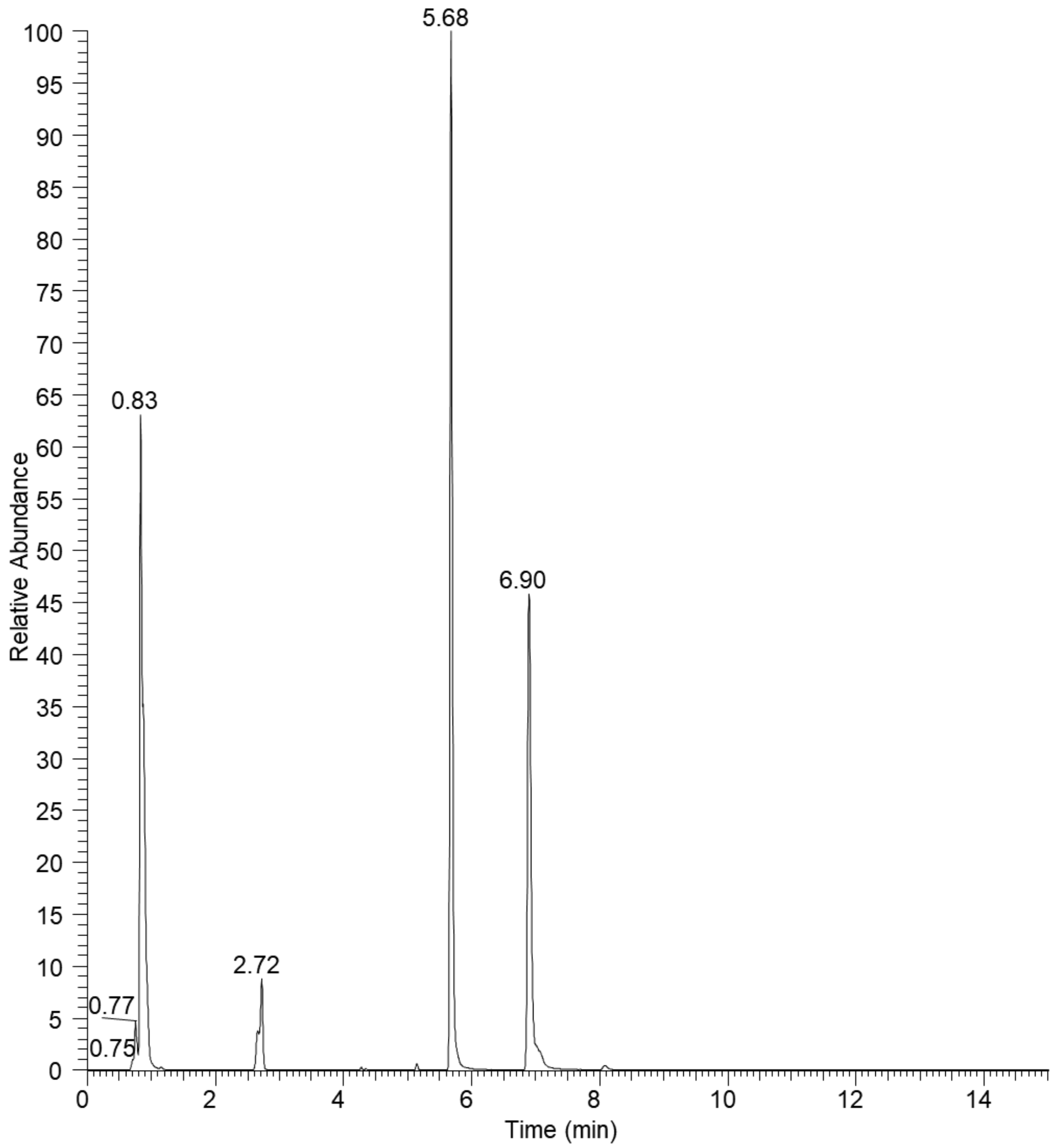

Figure S1: The mass spectrum of the drug group in the positive ion mode.

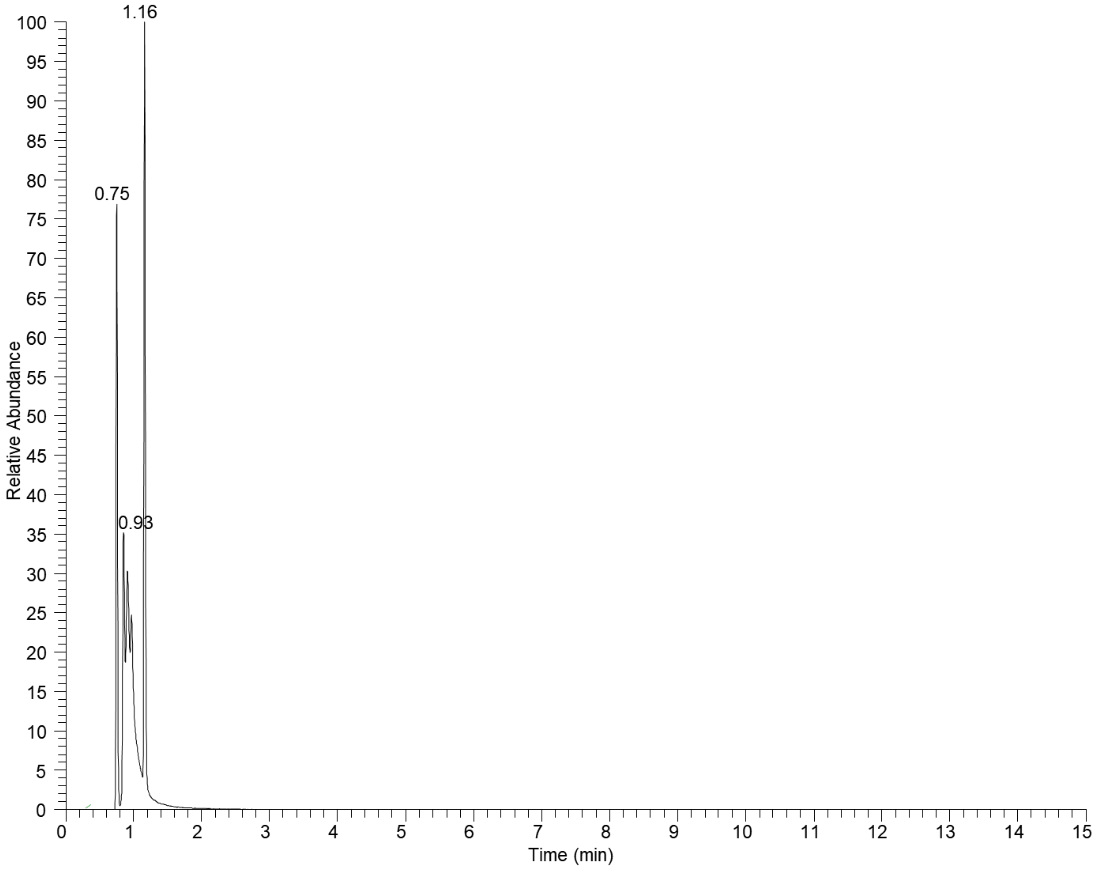

Figure S2:The mass spectrum of the drug group in the negative ion mode.
